# Brain-derived circulating endothelial cells in peripheral blood of newborn infants with seizures: a potential biomarker for cerebrovascular injury

**DOI:** 10.14814/phy2.12345

**Published:** 2015-03-24

**Authors:** Massroor Pourcyrous, Shyamali Basuroy, Dilyara Tcheranova, Kristopher L Arheart, Mohamad T Elabiad, Charles W Leffler, Helena Parfenova

**Affiliations:** 1 Department of Pediatrics, The University of Tennessee Health Science Center (UTHSC)Memphis, Tennessee; 2 Department of Physiology, The University of Tennessee Health Science Center (UTHSC)Memphis, Tennessee; 3 Department of Neuroscience Institute, The University of Tennessee Health Science Center (UTHSC)Memphis, Tennessee; 4 Department of Obstetrics and Gynecology, The University of Tennessee Health Science Center (UTHSC)Memphis, Tennessee; 5 Division of Biostatistics and Pediatrics, Department of Public Health Sciences, Miller School of Medicine, University of MiamiCoral Gables, Florida

**Keywords:** Asphyxia, BCECs, CECs, HIE, IVH, neonates

## Abstract

Neonatal seizures have been associated with cerebrovascular endothelial injury and neurological disabilities. In a piglet model, the long-term loss of endothelial regulation of cerebral blood flow coincides with the surge of brain-derived circulating endothelial cells (BCECs) in blood. We hypothesized that BCECs could serve as a noninvasive biomarker of cerebrovascular injury in neonates with seizures. In a prospective pilot feasibility study, we enrolled newborn infants with confirmed diagnoses of perinatal asphyxia and intraventricular hemorrhage (IVH); both are commonly associated with seizures. Infants without clinical evidence of cerebrovascular injuries were representative of the control group. BCECs were detected in the CD45-negative fraction of peripheral blood mononuclear cells by coexpression of CD31 (common endothelial antigen) and GLUT1 (blood-brain barrier antigen) via automated flow cytometry method. In Infants with asphyxia (*n* = 12) and those with IVH grade III/IV (*n* = 5), the BCEC levels were 9.9 ± 0.9% and 19.0 ± 2.0%, respectively. These levels were significantly higher than the control group (*n* = 27), 0.9 ± 0.2%, *P *< 0.001. BCECs in infants with cerebrovascular insults with documented clinical seizures (*n* = 10; 16.8 ± 1.3%) were significantly higher than infants with cerebrovascular insults with subclinical or no seizures (*n* = 7; 9.5 ± 1.2%); *P* < 0.001. BCEC levels decreased with seizure control. BCECs levels were elevated in infants with seizures caused by severe IVH and perinatal asphyxia. We suggest that monitoring BCEC levels in peripheral blood can potentially offer a biological marker that reflects cerebrovascular insult and recovery. Further studies with a larger number of patients are required to support these findings.

## Introduction

Seizures secondary to perinatal asphyxia and intraventricular hemorrhage (IVH) represent the most frequent neurological events in newborn infants, with increased risk of long-term neurological disabilities (Mizrahi 1999; Wasterlain et al. 2002; Ferriero 2004; Clancy 2006). Clinical data in human neonates indicate association of clinical seizures with cerebrovascular endothelial damage (Cornford 1999; Oby and Janigro 2006). In our studies in newborn piglets, we demonstrated the development of cerebrovascular endothelial dysfunction following epileptic seizures (Carratu et al. 2003; Parfenova et al. 2005), severe asphyxia (Pourcyrous et al. 1997a), ischemia (Leffler et al. 1989), and intracranial hemorrhage (Parfenova et al. 1993; Pourcyrous et al. 1997b). The likely consequences of cerebrovascular endothelial dysfunction are loss of cerebral blood flow auto regulation, inability to adequately adjust blood supply to the brain, and failure of blood-brain barrier (BBB) functions (Abbott and Friedman 2012).

There are limited noninvasive methods for early detection of cerebrovascular damage in clinical settings (Woywodt et al. 2012). Circulating endothelial cells (CECs), the cell components of peripheral blood mononuclear cells (PMNCs) have been proposed as diagnostic markers of endothelial damage caused by systemic vascular disease (Gaynor et al. 1970; Makin et al. 2004; Woywodt et al. 2004, 2012; Lee et al. 2005; McClung et al. 2005; Boos et al. 2006; Chong et al. 2006; Grundmann et al. 2008; Deb et al. 2013).

Our pioneering studies in newborn piglets have established relationships between cerebral vascular disorder and CECs of brain vessel origin, the BCECs. We have demonstrated that BCECs serve as a sensitive indicator of cerebral vascular endothelial damage and long-term cerebral blood flow dysregulation caused by epileptic seizures.

The purpose of the present translational study is, to investigate the relationship between BCECs, CECs, and human cerebrovascular injury. We hypothesize that in human neonates BCEC levels in peripheral blood increase with cerebrovascular injury.

## Methods

### Protocol

The protocol was approved by the Institutional Review Board of the University of Tennessee Health Science Center in Memphis, Tennessee. The study was carried out at the neonatal intensive care unit (NICU) at the Regional One Health. Written informed consent was obtained from the parents. When the newborn infant needed to have blood drawn for laboratory test, extra blood (0.5 to 1.0 mL) was collected for later analysis of BCECs. A preset limit of up to five blood samples could be obtained from an individual neonate during the NICU stay.

## Subjects

All newborn infants, regardless of sex, race, birth weight (BW), gestational age (GA), and postnatal age were eligible for this study. Specifically, we were interested in enrolling infants with cerebrovascular insults, such as perinatal asphyxia and IVH, both common causes of seizures. A clinical diagnosis of seizures was made by two senior medical personnel (one Neonatologist) by documenting the presence of generalized tonic-colonic jerky movements that was not controlled by gentle holding of the extremities that was accompanied by increased blood pressure, and heart rate and decreased oxygen saturation. Conventional EEG and/or amplitude integrated EEG were ordered as clinically indicated by the primary team. Newborn infants were designated as having perinatal asphyxia if they met three of the following criteria: (1) evidence or suspicion of hypoxic-ischemic brain injury based on a history of fetal distress; (2) need for resuscitation after birth; (3) base deficit of >15 mmol/L in cord blood or admission arterial blood sample; and (4) presence of abnormal neurologic signs, such as hypotonia/hypertonia or seizures in the immediate postnatal period (Pourcyrous et al. 1997b) Diagnosis of IVH was made by a Radiologist based on Papile scoring criteria (Papile et al. 1978). The control group included newborn infants without clinically obvious cerebrovascular insults. However, since these neonates were admitted to NICU, they had some other clinical conditions that necessitated their admission for observation or treatment; conditions such as rule out infection, respiratory distress, apnea of prematurity, chronic lung disease or poor oral feeding due to prematurity.

### Detection of BCECs and CECs

Blood samples were collected in K_3_-EDTA-containing Vacutainer tubes (BD, Franklin Lakes, NJ) and were processed within 2 h. PMNCs were separated by Histopaque 1077 density gradient centrifugation (Parfenova et al. 2010). PMNCs were suspended in the cryoprotectant medium (80% fetal bovine serum and 20% DMSO) and stored in a liquid nitrogen tank (Kleeberger et al. 1999; Disis et al. 2006). Cryopreservation of PMNCs preserves antigen expression for up to 12 years (Kleeberger et al. 1999).

Based on our previous experience using several endothelial-specific antigens for detection of BCECs in peripheral blood (Parfenova et al. 2010), we developed a time- and cost-effective protocol for BCECs detection using specific antigens by flow cytometry method. BCECs were detected by automated flow cytometry as CD45^−^/CD31^+^/GLUT1^+^ fraction of PMNCs based on three cell-specific antigens: CD45 (common leukocyte antigen), CD31 (common endothelial marker) and GLUT1 (BBB endothelial marker). CECs were characterized as the CD45^−^/CD31^+^ fraction of PMNCs. Cerebral microvascular endothelial cells (CMVEC) from newborn piglets were used as the positive control for BCECs. Human umbilical vein endothelial cells (HUVECs, VEC Technologies, Rensselaer, NY) were used as the positive control for CECs. CD45 and CD31 antigens were detected by direct staining using (M)CD45-PerCP-Cy5.5 and (M)CD31-PE (R-phycoerythrin), respectively (antibodies from BD Biosciences; San Jose, CA). GLUT1 was detected by indirect staining of Triton X-100-permeabilized PMNCs using (R) A/GLUT1 (EMD Millipore, Billerica, MA) and goat A/R IgG-FITC (BD Biosciences).

### Outcome at the time of discharge from the hospital

Abnormal neurological outcome was defined as in-hospital death or abnormal neurologic findings, such as abnormal muscle tone (moderate to severe hypotonia or hypertonia), uncoordinated or absent sucking and swallowing reflexes inappropriate for the gestational age, or the need to continue anticonvulsant medicine.

### Statistical methods

**Figure 1:**
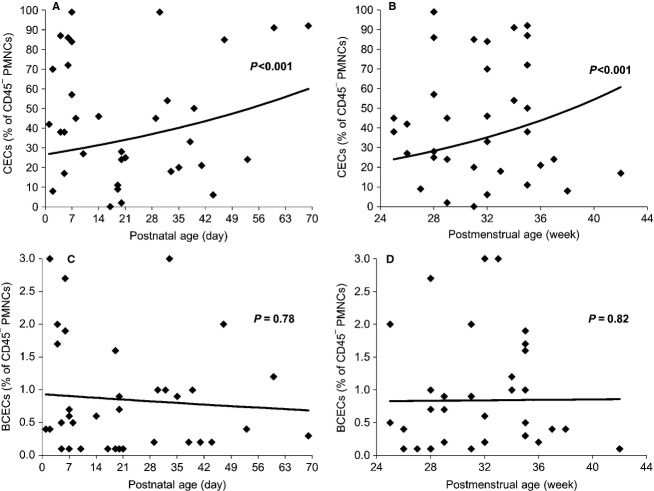
CECs (A and B) and BCECs (C and D) are plotted against the postnatal age (days) and postmenstrual age (weeks) in control infants (preterm). There was a significant increase in CECs values with postnatal and postmenstrual age in the control group. However, BCECs remained at low levels of <1%.

Logistic regression for binary outcomes (sex and race), one-way analysis of variance for continuous outcomes (BW, GA, and age at first measurement), and Poisson regression for cell counts for first blood samples were used to analyze the data presented in Table1. For all regression analyses, Poisson regression provided the best fit for the data, as opposed to linear regression for normally distributed data. Planned comparisons via *t*-test were made between the control group and all others. Generalized linear mixed model analysis for Poisson regression was used to calculate the regression lines for the Fig.1A–D. The model included fixed effects for group (control, asphyxia/HIE, IVH grade II, and IVH grades III/IV) and time (postnatal or postmenstrual as noted in text and figures) nested within group. A random intercept term was included with subjects within group to control for repeated measurements. Significance tests for intercepts, slopes, and regression equations were made with planned contrasts. Spearman correlation was used to assess the association of BW with BCECs and CECs. Poisson regression was used to compare CECs and BCECs between babies with and without clinical seizures. *P* < 0.05 was considered significant. Data are presented as frequencies (%), range, mean ± SEM, or median (Q1–Q3) as appropriate. sas 9.3 (SAS Institute Inc.; Cary, NC) was used for all analyses.

**Table 1:** Characteristics of all infants by group (*n* = 51)

Group (*n*)	Control (27)	IVH II (7)	IVH III/IV (5)	Asphyxia (12)
Black (*n*)	22 (81%)	7 (100%)*	3 (80%)	5 (42%)*
Male (*n*)	12 (44%)	5 (71%)	3 (60%)	5 (42%)
Birth weight (g)	1130 ± 113 928 (680–1165)	804 ± 223 805 (654–860)	691 ± 264 670 (652–685)	2811 ± 171* 2651 (2126–3277)
Gestational age (week)	28 ± 1 27 (25–31)	26 ± 1 26 (25–27)	25 ± 2* 25 (24–26)	36 ± 1* 36 (35.5–37.5)
Clinical seizures (*n*)	0	0	4	6
Infant with more than one sample (*n*)	5	5	4	7
Total blood samples (*n*)	35	20	16	30
Age at blood samples (d)	1–69	7–104	3–90	2–21
Age at 1st blood sample (d)	21 ± 3 17 (5–35)	15 ± 6* 16 (8–20)	7 ± 7* 6 (5–7)	5 ± 4* 2.5 (2–6.5)
BCECs (% of CD45^−^ PMNCs) at 1st blood sample	0.9 ± 0.2 0.6 (0.2–1.6)	1.2 ± 0.4 1.2 (0.4–1.7)	19.1 ± 2.0* 12.9 (7.3–37)	9.9 ± 0.9* 4 (2.3–24.5)
CECs (% of CD45^−^ PMNCs) t 1st blood sample	44.9 ± 1.3 38 (17–85)	55.6 ± 2.8* 56 (27–95)	42.6 ± 2.9 47 (46–53)	53.4 ± 2.1* 52 (26–76)

IVH, intraventricular hemorrhage; d, days; *n*, numbers; BCECs, brain-derived circulating endothelial cells; CECs, circulating endothelial cells; PMNCs; peripheral blood mononuclear cells.

Statistical analysis: Values are presented as frequency (%), range, mean ± SEM, or Median (Q1–Q3) as appropriate. *P < 0.05 is considered significant. Comparisons were made with the control group. Logistic regression was used for race and gender. One-way ANOVA was used for birth weight, gestational age, and age when the 1st blood sample was obtained. Poisson regression was used for BCECs and CECs.

## Results

### Characteristics of infants

Table1 represents characteristics of all groups (control, IVH grade II, IVH grade III/IV and asphyxia).

### CEC values in different groups

We analyzed whether the number of CECs in the control group changed with postnatal or postmenstrual age. Postmenstrual age was defined as GA at the time of delivery plus postnatal age at the time of blood withdrawal (*n* = 27 infants; *n* = 35 blood samples; Fig.1A and B). CECs increased with postnatal age (intercept = 3.28 ± 0.19, *P *<* *0.001; slope = 0.012 ± 0.005, *P *=* *0.021; regression equation *P *<* *0.001), and with postmenstrual age (intercept = 1.82 ± 0.90, *P *=* *0.05; slope = 0.05 ± 0.03, *P *=* *0.05; regression equation *P *<* *0.001). Additionally, there was no association between the BW and the CECs (*r* = −0.119, *P *=* *0.41). CECs values in all groups are presented in Table1.

CECs in infants with severe IVH or asphyxia who had documented clinical seizures (*n* = 10) were lower (45.0 ± 2.1%) compared to those infants with cerebrovascular insults who had no seizures or subclinical seizures (*n* = 7; 65.7 ± 3.0%; *P* < 0.001).

### BCEC values in infants by group

#### Control

BCECs were mostly undetectable or detected at very low numbers (0.9 ± 0.2%; Table1). BCEC levels did not change with postnatal age (intercept = −0.07 ± 0.40, *P *=* *0.087; slope = −0.005 ± 0.014, *P *=* *0.78; regression equation *P *=* *0.78), or with postmenstrual age (intercept = −0.24 ± 2.25, *P *=* *0.92; slope = 0.002 ±0.07, *P *=* *0.98; regression equation *P *=* *0.82; Fig.1C and D). Additionally, there was no association between BW and the BCECs values (*r* = 0.107, *P *= 0.46). Twelve samples were collected in the first week of life. BCEC levels were similar to those collected after 7 days of life (mean 1.178 ± 1.01; median 0.65 (0.4–1.98), *P* > 0.05).

#### IVH grade II

In newborn infants with IVH grade II the numbers of BCECs were low and comparable to the control group (1.2 ± 0.4%; *P *=* *0.550; Table1). We did not find any significant variations in the BCECs numbers during the hospital stay (intercept = 0.14 ± 0.59, *P *=* *0.81; slope = −0.015 ± 0.016, *P *=* *0.37; regression equation *P *=* *0.59).

#### IVH grade III/IV

**Figure 2:**
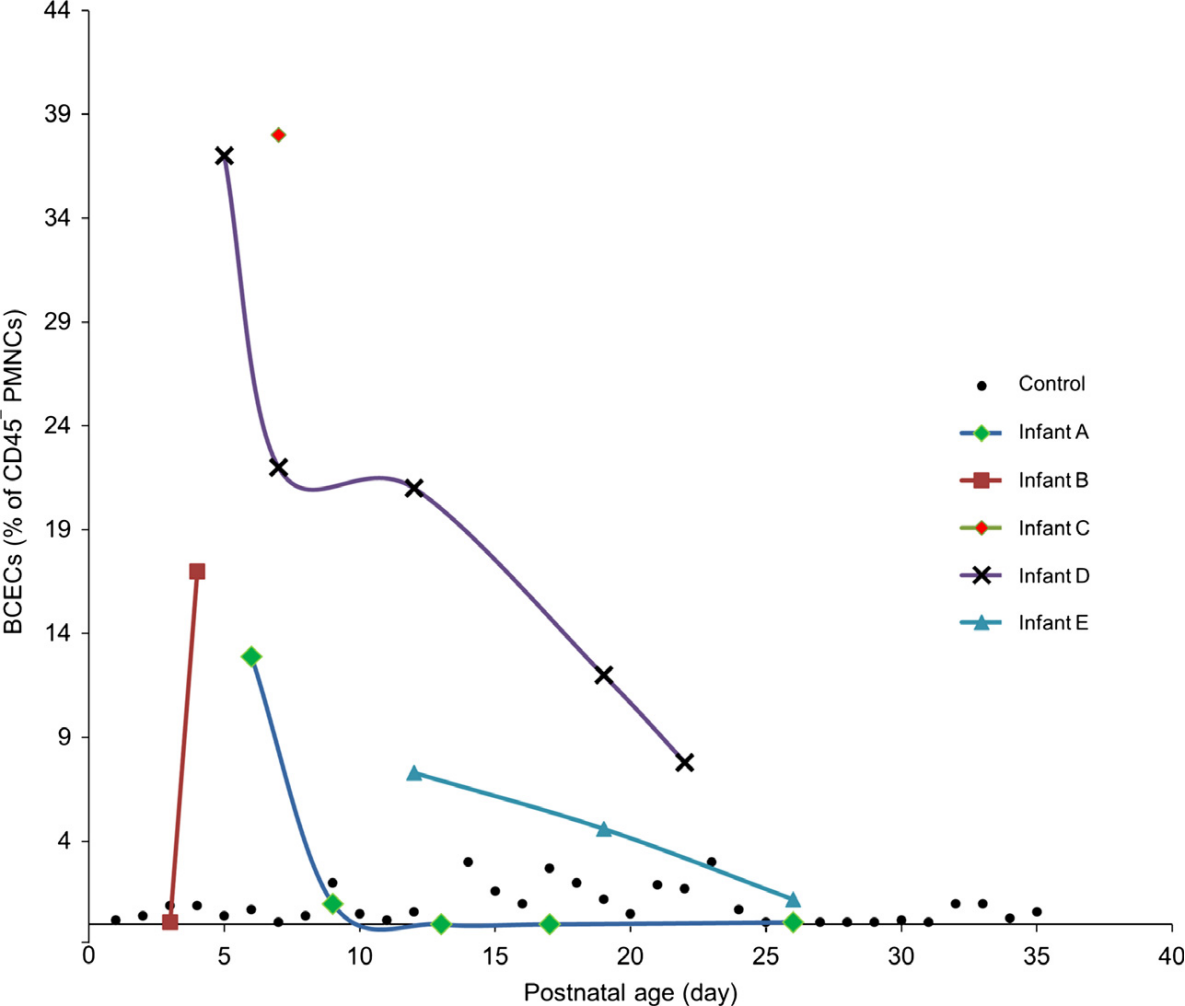
BCEC values (as the percentage of CD45^−^/CD31^+^/Glut1^+^ fraction of PMNCs) obtained over time from each infant with IVH grade III/IV plotted against the postnatal age (d). The first sample was collected at or as close as possible to the time of clinical manifestation of the cerebrovascular insult (*N* = 5 infants; *N* = 16 blood samples). Each infant is presented with a different symbol and color. Control infants are plotted for comparisons (*N* = 27 infants; *N* = 35 blood samples). BCECs, brain-derived circulating endothelial cells; PMNCs, peripheral blood mononuclear cells

**Figure 3:**
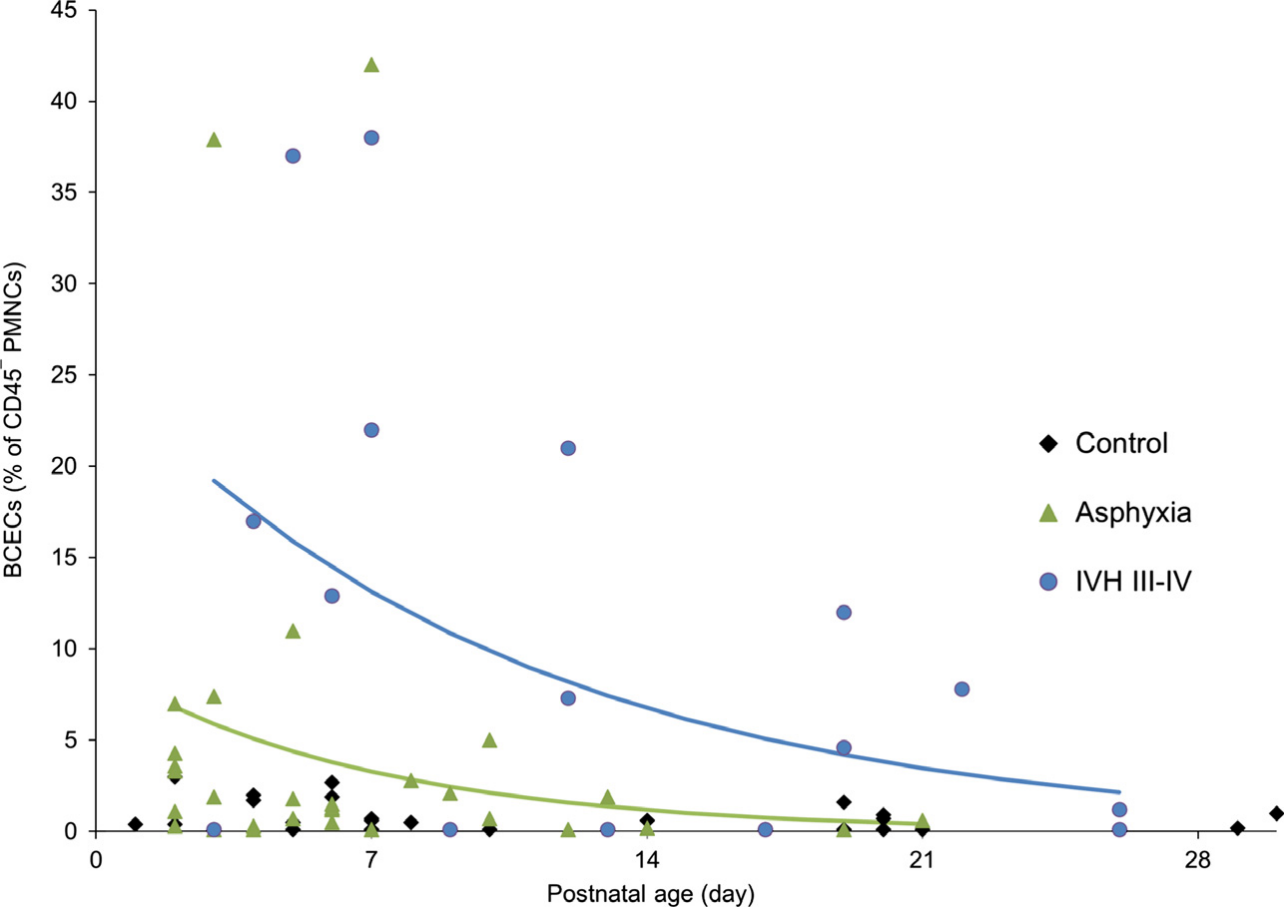
BCECs in infants with cerebrovascular insults (asphyxia and IVH grade III/IV) plotted against the postnatal age (days). The first sample was collected at or as close as possible to the time of clinical manifestation of the cerebrovascular insult. The asphyxia group, *N* = 12 infants; *N* = 30 samples. IVH grade III/IV group, *N* = 5 infants; *N* = 12 samples. BCECs in control infants are plotted for comparisons; *N* = 27 infants; *N* = 35 blood samples. BCECs values are elevated in infants with cerebrovascular insults, but decreased with frequent measurements.

The newborn infants with IVH grade III/IV had significantly elevated BCECs compared to the control group (19.1 ± 2.0%; *P *<* *0.001; Table1). Figure2 represents changes in BCECs in individual patient over time; the first blood sample was collected close to the time of clinical seizures. BCECs decreased with follow-up measurements (intercept = 3.24 ± 0.46, *P *<* *0.001; slope = −0.095 ± 0.016, *P *<* *0.0001; regression equation *P *<* *0.001 (Fig.3). In one infant with grade IV IVH who had drug-resistant clinical seizures, BCECs remained elevated up to 17 day, the day the last sample was obtained (Fig.2).

#### Asphyxia

In the asphyxia group the BCECs were elevated 10 times above the control group (9.9 ± 0.9%; *P *<* *0.001; Table1). In six infants, BCECs were high on first sample but decreased over time when multiple blood samples were obtained. In remaining infants BCECs remained at low levels (<1%) during the observational period. In many infants the time of the asphyxia insult could not be determined. We observed that if the blood sample was obtained within 2 days of acute asphyxia insult, such as an abruptio placentae event, the BCECs would be high. However, if the asphyxia insult occurred several days prior to the delivery of the infant (maternal report of decreased fetal movement) BCECs would remain at a low level. BCECs values in newborn infants with asphyxia decreased during their hospital stay (intercept = 2.2 1 ± 0.38, *P *<* *0.001; slope = −0.146 ± 0.042, *P *=* *0.001; regression equation *P *<* *0.001) (Fig.3).

#### BCECs and short-term neurological outcome of infants with cerebrovascular insults (IVH grade III/IV and Asphyxia) with or without documented clinical seizures; *n* = 17)

In 10 infants who had documented clinical seizures, the BCECs value was significantly higher than the infants without documented clinical seizures (*n* = 7) (16.8 ± 1.3% and 9.5 ± 1.2%, respectively; *P *=* *0.001).

In seven of those ten infants, seizures were also confirmed by an EEG. Overall, infants with documented clinical seizures and higher BCECs values had poor neurological outcomes during their hospital stay. Four infants either had a do not resuscitate (DNR) order or died. The remaining six infants still required anticonvulsant medications at the time of discharge. Two infants required gastrostomy-tube placement and three infants required ventriculoperitoneal shunt placement.

Six infants with diagnoses of asphyxia and one infant with grade III IVH did not have documented clinical seizures. However, EEGs obtained in three asphyxiated infants were reported as abnormal (subclinical seizures). One infant had DNR order for severe asphyxia, but the other six infants appeared neurologically normal at the time of discharge.

**Figure 4:**
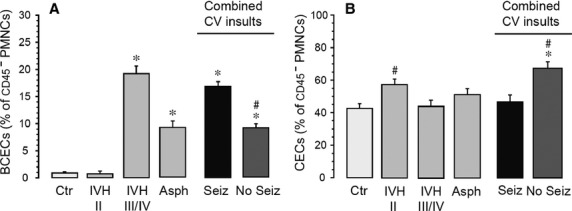
(A and B) Detection of BCECs (A) and CECs (B) in infants with and without cerebrovascular insults (CV insults) of different etiology. *P < 0.05 as compared with the control group; ^#^*P* < 0.05 as compared with the clinical seizures group.

Figure4 represents BCECs and CECs values in all groups.

## Discussion

Our study for the first time has identified brain-derived endothelial cell components of the peripheral blood (BCECs) as an early biomarker of cerebrovascular injury in human neonates with potential relevance to the pathophysiology and prognosis. The new findings of this study in newborn infants are: (1) the number of BCECs in the peripheral blood of infants without cerebrovascular insults is low or undetectable; (2) the number of BCECs is greatly increased in infants with acute asphyxia or severe IVH; and (3) the number of BCECs detected in infants with cerebrovascular insults who had documented clinical seizures were significantly higher than those with no or subclinical seizures; infants with higher levels of BCECs had poor short-term neurological outcomes. A relation between CECs levels and cerebrovascular insult could not be found.

The endothelium is important in regulating vascular contractility and vascular barrier properties (Abbott and Friedman 2012). Endothelial cells cover the vascular tree and adhere to the basement membrane. In health, these cells would be expected to stay in this location, with some cell loss into the blood, with ultimate clearance by the reticuloendothelial system (Woywodt et al. 2006). The mechanism of endothelial cell damage resulting in cell detachment from the basement membrane involves many factors, including mechanical injury, defective binding to anchoring matrix protein, and apoptosis (Blann et al. 2005; Woywodt et al. 2006; Parfenova et al. 2010). Experimental data in animals and clinical evidence in adult patients indicate vascular damage/dysfunction caused by diverse cardiovascular, inflammatory, and infectious insults correlates with the increased CECs values in peripheral blood (Mutunga et al. 2001; Makin et al. 2004; Furstenberger et al. 2005; Nadar et al. 2005; Boos et al. 2006; Li et al. 2013).

The normal level of CECs in infants is not known. We are reporting now that in preterm infants with no obvious signs of cerebral vascular disease (control group), CECs composed the majority of the leukocyte-depleted fraction of PMNCs (44.9 ± 1.3%). CECs in our preterm infants increased with postnatal age (up to 70 days) and also with postmenstrual age (up to 42 weeks). Various vascular sources can contribute to the appearance of CECs in peripheral blood of newborn babies. In addition to complications related to the prematurity that involves multiple organ systems, a majority of infants in NICU have intravascular catheter placement that may cause endothelial damage leading to the appearance of CECs in blood.

In infants with IVH grade III/IV, the number of CECs was not significantly higher from the control group (42.6 ± 2.9%). Also, infants with cerebrovascular insults who had poor neurological outcome had lower CECs compared to those with normal neurological outcome at the time of discharge (45.0 ± 2.1 and 65.7 ± 3.0, respectively; *P* < 0.001). Unlike adults, we could not find an association between changes in CECs values and the severity of cerebrovascular insults.

Previously we demonstrated in newborn piglets the development of cerebrovascular endothelial dysfunction following epileptic seizures, (Carratu et al. 2003; Parfenova et al. 2005) severe asphyxia, (Pourcyrous et al. 1997a) ischemia, (Leffler et al. 1989) and intracranial hemorrhage (Parfenova et al. 1993; Pourcyrous et al. 1997b). The likely consequences of cerebrovascular endothelial dysfunction are loss of cerebral blood flow auto regulation, inability to adequately adjust blood supply to the brain, and failure of blood-brain barrier (BBB) functions (Abbott and Friedman 2012). Also, experimental findings in newborn piglets demonstrated that seizure-induced BCECs are injured ECs dislodged from the cerebral micro vessels (Parfenova et al. 2010). However, the relationships between the cerebrovascular insult and the appearance of BCECs in human beings, in particular, have not been described previously. Our study demonstrated that BCECs are greatly increased in infants with cerebrovascular insults as a result of asphyxia and severe IVH.

Importantly, the higher numbers of BCECs were observed in infants with cerebrovascular insults who had documented clinical seizures rather than subclinical seizures; these infants also had poor neurological outcome. Cerebral blood flow (CBF) disturbance is more pronounced when it is accompanied by clinical seizures compared to subclinical seizures (Pourcyrous et al. 1992). Also, clinical data in human neonates indicate association of clinical seizures with cerebrovascular endothelial damage (Cornford 1999; Oby and Janigro 2006). We speculate that clinical seizures that are commonly accompanied by significant increase in blood pressure, hypercapnia, hypoxia, and acidosis will result in more cerebrovascular endothelial dysfunction and more BCECs release in peripheral circulation. Also, highest values of BCECs in neonates with asphyxia and IVH were observed when blood was collected close to the time of insult; this was specifically documented in infants with severe IVH.

Limitations of this pilot study include: (1) small number of term and white infants in the control group; (2) a limited number of infants with cerebrovascular insults; (3) the time-points of sample collections were not consistent due to the restriction for the blood draw imposed in the consent form, and (4) a lack of long-term neurological follow-up for those infants that presumably had normal short-term neurological findings. However, these limitations do not reduce the validity of our findings, because we observed a significant correlation between the severity of cerebrovascular insult, and the number of BCECs in peripheral blood.

This is the first clinical study on detection and clinical significance of BCECs in neonates with cerebrovascular insult. BCECs levels were elevated in infants with seizures caused by severe IVH and perinatal asphyxia. We suggest that BCECs may serve as valuable indicators of acute or ongoing cerebral vascular damage in newborns. It is therefore important to collect blood as early as an insult is suspected and to follow BCEC levels over time to evaluate progression of disease and response to treatment as in the case of clinical seizures. We propose a larger prospective study that delineates normal BCEC levels by gestational age and postnatal age and how these levels may change in conditions associated with cerebrovascular injury and in response to initiation of treatment.
